# Competing priorities and second chances - A qualitative exploration of prisoners’ journeys through the Hepatitis C continuum of care

**DOI:** 10.1371/journal.pone.0222186

**Published:** 2019-09-11

**Authors:** Desmond Crowley, Walter Cullen, John S. Lambert, Marie Claire Van Hout

**Affiliations:** 1 Irish College of General Practitioners, Lincoln Place, Dublin, Ireland; 2 School of Medicine, University College Dublin, Dublin, Ireland; 3 Department of Infectious Diseases, Mater Misericordiae University Hospital, Dublin, Ireland; 4 Liverpool John Moores University, Liverpool, United Kingdom; Macfarlane Burnet Institute for Medical Research and Public Health, Australia, AUSTRALIA

## Abstract

High levels of undiagnosed and untreated HCV infection exist in prison populations globally. Prisons are a key location to identify, treat and prevent HCV infection among people who inject drugs (PWID). Understanding prisoners’ lived experiences of the HCV continuum of care informs how HCV care can be effectively delivered to this marginalised and high-risk population. This study aimed to explore Irish prisoners’ experience of prison and community-based HCV care. We conducted one-to-one interviews with 25 male prisoners with chronic HCV infection. Data collection and analysis was informed by grounded theory. The mean age of participants and first incarceration was 39.5 and 18.3 years respectively. The mean number of incarcerations was eight. The following themes were identified: medical and social factors influencing engagement (fear of treatment and lack of knowledge, HCV relevance and competing priorities), adverse impact of HCV on health and wellness, positive experience of prison life and health care and the transformative clinical and non-clinical changes associated with HCV treatment and cure. Findings suggest that prison release was associated with multiple stressors including homelessness and drug dependence which quickly eroded the health benefits gained during incarceration. The study generated a substantive theory of the need to increase the importance of HCV care among the routine competing priorities associated with the lives of PWID. HCV infected prisoners often lead complex lives and understanding their journeys through the HCV continuum can inform the development of meaningful HCV care pathways. Many challenges exist to optimising HCV treatment uptake in this group and incarceration is an opportunity to successfully engage HCV infected prisoners who underutilise and are underserved by community-based medical services. Support and linkage to care on release is essential to optimising HCV management.

## Introduction

High levels of undiagnosed and untreated Hepatitis C Virus (HCV) infection exist in prison populations globally[1–3]. As a result of the ongoing criminalisation and imprisonment of people who inject drugs (PWID), the main risk factor for HCV infection in prisoners is injecting drug use (IDU)[1,2,4]. An estimated 25% of the global prison population has been exposed to HCV[1]. Prison is a critical setting to access HCV infected PWID and provides an important public health opportunity to manage this epidemic.

HCV screening and treatment in PWID and prisoners is low (9%-12%)[5–7]. Barriers and enablers to engagement in the HCV care continuum have been identified in both groups[8–10]. Reducing the pool of HCV infection in both prisoners and PWID requires increasing the proportion engaging in all aspects of the HCV care cascade and understanding the challenges to screening, linkage to care, treatment and prevention experienced by those infected. The availability of highly effective and tolerable direct-acting antiviral (DAA) treatments have made HCV infection curable and have removed many of the barriers associated with interferon-based treatment regimens [11,12].

Many jurisdictions are scaling up HCV screening and treatment in PWID and prisoners with a view to achieving the WHO target of eliminating HCV infection as a major public health concern by 2030[11,13,14]. Evidence has demonstrated that “treatment as prevention” in both groups is cost-effective and an achievable HCV public health strategy[7,15,16]. Studies are reporting near or micro-elimination of HCV infection in certain communities and prison settings[17–19]. Despite having the tools to cure and prevent HCV infection and the optimism and enthusiasm brought by these reported successes, many countries, including Ireland, struggle to expand HCV care to prisoners. There are no Irish prison-based needle syringe programmes (NSP) but methadone maintenance treatment (MMT) is available at all prison locations in the Republic of Ireland.

There are over 3,500 persons incarcerated in Ireland on any given day with an annual turnover of over 7,500 unique prisoners[20]. The most recent national estimate of HCV prevalence in Irish prisoners is 13% with significant inter-prison variation related to levels of PWID incarcerated at the prison location[21]. Recently published national screening guidelines recommend HCV screening for all prisoners and national HCV treatment guidelines support the treatment of all HCV infected patients with DAA[22,23]. In-reach hepatology services are provided at three of the 12 Irish prisons. The Irish National Hepatitis C Treatment Programme (NHCTP) have identified prisons as key locations to identify and treat those who have not been referred to, or engaged with community HCV care[24].

A small number of studies have reported on the lived experience of HCV infected PWID in the community[25–27]. Previous studies have reported on barriers, enablers and motivators to prisoners’ engagement with HCV care[28–30]. To our knowledge this is the first study to report on HCV infected prisoners’ experience of the entire HCV continuum of care, from infection to cure, both in the community and prison. The study aims to collect and analyse prisoners’ narratives of their HCV journey which will inform HCV care delivery to this marginalised and underserved group, nationally and internationally.

## Materials and methods

This study is reported according to the Consolidated criteria for Reporting Qualitative research (COREQ). Ethical approval was obtained from the Mater Ethics Committee as part of the Seek and Treat component of The European Hep Care Project and supported and endorsed by the Irish Prison Service’s Ethics Review Committee[31].

One-to-one, in-person and in-depth semi-structured interviews were conducted in a purposeful sample of 25 prisoners identified as having untreated active HCV infection and offered DAA treatment during their current prison sentence. Sampling was purposeful aiming to maximise variation in age, country of origin, liver disease stage, HIV co-infection, stage of treatment including treatment deferral (DAA) and non-completion (interferon-based). Three selected prisoners refused to participate, two due to family visits and one due to a court appearance. Data was collected from July-November 2018 at Mountjoy Prison, an all-male medium-security prison located in Dublin, Ireland. A data collection tool (Tables 1 and 2) and interview guide (S1 File) was designed by the research team and included questions related to all parts of the HCV treatment cascade including HCV acquisition risks. The interview guide covered the following themes: HCV journey in the community (risk behaviour, diagnosis and engagement in HCV care), barriers to community HCV engagement, impact of HCV infection on health and quality of life, description of prison HCV journey (both current and previous incarcerations), the experience of treatment, the impact of treatment on health and wellness, concerns about re-infection and the positives and negatives of prison based HCV care. Informed consent was sought from interviewees prior to study participation. Participants were assured of confidentiality, anonymity and the ability to withdraw from the study should they wish. Prisoners were reassured that refusal to participate or their withdrawal from the study would not impact their medical care. No financial inducement for study participation was offered. The interviews were conducted by the lead author DC, in a private consultation room at Mountjoy Prison and lasted an average of 35 minutes each. DC is the addiction specialist doctor at the study location and was known and provided clinical care to all the research participants. Demographic data was collected on a standardised data collection form by the interviewer prior to starting the in-depth interview. Digital recordings of the interviews were made and were later transcribed verbatim for data analysis and destroyed following transcription. Transcripts were coded for anonymity and following the removal of any personal identifiers stored on a password-protected encrypted hard drive. Demographic data was anonymised and uploaded to an encrypted Excel sheet using Microsoft word. All participant data was stored in accordance with Irish and European data protection laws. Statistical analysis on collected demographic data was performed using the Statistical Package for Social Science (verison23). Data is primarily expressed as means (SD) or n (%).

**Table 1:** Description of study participants.

Pseudonym	Age	Treatment status
Duane	42	Completed treatment with SVR
Raymond,	42	Completed treatment with SVR
Anthony	46	Deferred treatment
Shane	38	HIV co-infected and completed treatment with SVR
David	42	On treatment
Paul	52	Completed treatment with SVR
Noel	33	Deferred treatment
Dean	36	Completed treatment awaiting SVR
Gavin	37	On treatment
Michael	35	Deferred treatment
Brendan	59	Completed treatment with SVR
Nathan	40	HCV co-infected and completed treatment
Edward	38	On treatment
Gary	30	Completed treatment with SVR
Thomas	35	Completed treatment with SVR
William	38	On treatment
Patrick	44	Completed and awaiting SVR
Daniel	41	On treatment
John	47	HIV co-infected on treatment
Clint	36	Completed treatment with SVR
Kenneth	41	Completed treatment awaiting SVR
Colm	38	Completed treatment with SVR
Graham	34	Completed treatment with SVR
Emmett	41	Completed treatment with SVR
Leslie	42	On treatment

SVR: Sustained virological response (HCV RNA negative 12 weeks post DDA treatment); HCV: Hepatitis C virus; HIV: Human Immunodeficiency Virus

**Table 2:** Demographics of study participants in Mountjoy Prison, Ireland (September 2018).

Variable	Value n	Value %	Mean (SD)
**Age**			39.5 (6.4)
**Age of first incarceration**			18.3 (6.8)
**Episodes of incarceration**			8 (2.6)
**Total time incarcerated (years)**			12.5 (5.8)
**Age of first drug use**			15. 3 (3.2)
**Age of first Injecting drug use**			18.5 (3.2)
** Country of origin**			
**Ireland**	23	92	
**HCV acquisition risk factors**			
**History of heroin use**	25	100	
**History of injecting drug use**	25	100	
**Shared needles (syringes) in the community**	18	72	
**Shared equipment (drug taking paraphernalia) in the community**	25	100	
**Shared razor in prison(ever)**	2	8	
**Shared toothbrush in prison(ever)**	2	8	
**Prison tattoo**	8	32	
**Unsterile tattoo community**	2	8	
**Self-declared alcohol use**			
**Alcohol problem prior to incarceration**	8	32	
**Treatment for alcohol use**	1	4	
**Methadone maintenance treatment (community and prison)**			
**Methadone treatment history**	18	72	
**Length of time on methadone maintenance treatment**			9.2 (5.8)

Values are means (±SD) for continuous variables or n (%) for categorical variables

Transcripts were imported into QSR International’s NVivo 11 software (NVivo qualitative data analysis Software 2012) for coding and thematic analysis following a grounded theory approach [32,33]. This approach was chosen because prisoners are considered a hard-to-reach population and their experiences of HCV care are poorly understood and rarely reported in the published literature [34,35].

Thematic coding comprised four stages, data familiarisation, initial low-level indexing of collected data, second level line-by-line open coding concentrating on the generation of data-driven categories and subcategories and finally focused coding involving refining and mapping second level codes within and across categories[33]. The thematic coding was revised with each analysis of the interview transcripts and data collection and analysis ceased when thematic saturation was achieved (agreed by researcher 1 and 2). Illustrative quotes from the interview transcripts supporting the thematic analysis are included and are reported using an assigned pseudonym and the participant’s age.

## Results

A total of 25 male prisoners participated (Table 1). The mean age of the study population was 39.5 years. Participants were incarcerated from an early age (mean = 18.3 years), experienced multiple incarcerations (mean = eight) and spent over a decade in prison (mean = 12.5 years). All participants reported a history of IDU. Two patients were co-infected with HIV infection, 10 had completed treatment with sustained virological response (SVR), three completed treatment and were awaiting 12-week post-treatment bloods (i.e. SVR12), seven were on treatment and two had deferred treatment at the time of their interviews.

Two participants had a period of community release during their current sentence (revoked temporary release (TR)) and their experiences of community release are included in the narratives. All participants had a history of sharing drug-taking paraphernalia in the community. 72% of the study cohort had a history of MMT and had spent almost a decade (9.2 years) on this treatment. (Table 2)

The following themes emerged from analysis of the data: medical and social factors influencing engagement in HCV care in the community, adverse impact of HCV on health and wellness, positive experience of prison life and health care, the transformative clinical and non-clinical changes associated with HCV treatment and cure and the reality of community release. The study generated a substantive theory of the need to increase the importance of HCV care among the routine competing priorities associated with the lives’ of PWID.

### Medical and social factors influencing engagement in community HCV care

#### Fear of treatment, lack of knowledge, lack of relevance and competing priorities

Participants described many barriers to engagement with HCV care in the community. Many had witnessed friends struggling with historical treatment regimens and a number of those interviewed had discontinued interferon-based treatment due to the adverse side effects experienced.

“*I’d heard a lot of horror stories about symptoms and all that*, *side effects”*. (Gavin, 37, on treatment)

“*The bleeding nightmares and sweats were really*, *really bad* … *was on it for a few… a couple of weeks in 2001 and then I just stopped”*. (Colm, 38, completed treatment with SVR)

The fear of treatment was linked to a lack of knowledge with many participants still associating treatment with interferon-based therapies and related side effects. Many described not being aware of the new treatment options until being offered them in prison and some expected that injections were still needed for treatment.

“*They say knowledge is power*. *I didn’t know*. *I thought you had to inject yourself…afraid of the needle buzz and all that*, *couldn’t believe it was only a tablet”*. (Emmett, 41, completed treatment with SVR)

A consistent theme emerging from prisoners’ memories of their first diagnosis was one of relief when associated with a negative HIV diagnosis. For co-infected prisoners their HCV diagnosis became irrelevant when compared to their HIV diagnosis. Most participants were diagnosed with HIV in the late eighties/early nighties: a time when HIV was potentially a terminal diagnosis and when HCV infection was viewed as the less serious infection. Many viewed HCV as benign and lacking relevance and did not attribute any physical or mental health issues to being chronically infected until they had experienced the benefits of treatment.

“*I was in there* (prison) *and I got it* (BBV screen) *done and it was* …*the best Christmas present I ever got*. *Like I didn’t even mind that I had it* … *at that stage I was just thinking HIV”*. (Kenneth, 41, completed treatment awaiting SVR)

All participants describe their periods out of prison as stressful requiring a daily prioritisation of demands. Active dependence was often the priority demand. The struggle to treat withdrawals and manage cravings was overwhelming for most and was often the first and most pressing daily demand. While most where engaged in MMT, active “chaotic” drug use was common. Often re-incarceration was associated with criminal activity related to drug use and all participants described a revolving door of release and re-incarceration.

“*I was strung out when I came in …They asked me about it (HCV treatment) once or twice but I was*, *to be honest* … *my life was chaotic*, *I couldn’t manage it”*. (William, 38, on treatment)

### Adverse impact on health and wellness

Many participants described the negative impact HCV infection had on their physical and mental health. Symptoms included low energy and mood, fatigue, generalised aches and pains, distended abdomen, poor sleep and pale or yellow skin pallor. Often the severity of these symptoms was only recognised post-treatment.

“*I had no energy*, *that was one of the main things* …*I always had low energy*. *Pains; I used to get pains in the front of me stomach and in me liver*. *Sweats; I used to get sweats*. *My sleep wasn’t great either*, *it was on and off*. *And*, *I used to go yellow*, *people would say to me “I’m tanned” or “you’re real yellow”*. *And it was like that throughout the years”*. (Clint, 36, completed treatment with SVR)

A small number of participants described having a fatalistic view of their illness and continued high-risk drug taking based on this view. This fatalism was often as a consequence of experiencing family members or close friends becoming ill or dying of the disease.

“*The only reason why I kept on using needles is because I said to myself that in my head*, *I had it*. *I had hepatitis anyway*, *so it’s not going to make a difference*, *you know what I mean*. *I’m going to die- I thought I was going to die from hepatitis*, *because my sister died from hepatitis”*. (Patrick, 44, completed and awaiting SVR)

Many also described the stress and fear of transmitting the infection to their sexual partners, family and social contacts. For some, this prevented them entering long-term relationships. This often exacerbated existing shame and stigma.

“*It affected everything*, *even relationships when I was with a girl*. *I never told anybody that I had it*, *you know what I mean*. *And when I’d be with a girl*, *I’d always wear condoms*, *and when I was in the relationship for a while*, *she would say to me*, *“you don’t have to use condoms*. *I know you”*. *But I never told her*, *you know what I mean*, *I always used them”*. (Patrick, 44, completed and awaiting SVR)

“*It’s not something you want to tell your girlfriend that you have*, *and it’s not something that you want to pass on to her either* … *it stresses you out*.*”* (Noel, 33, deferred treatment)

All study participants described feeling stigma and shame associated with HCV infection. Various terms were used to illustrate this including: “feeling dirty”, “feeling manky” and “contaminated”. HCV associated stigma was mainly related to its association with IDU. Often this stigma continued after successful treatment with SVR since many participants associated being HCV antibody positive with still being infected and linked to historical IDU. Closely linked with stigma was fear of breach of confidentiality. The consequence of this fear was that participants only revealed their status to a few people (mainly close family members) which often reduced their support networks.

“*When I was diagnosed with hep C* … *I was ashamed* … *and then anywhere I’d like deny it* (HCV infection) *for a long time*. *I haven’t got hep C it was just something in the background that was just always there”*. (Gavin, 37, on treatment)

“*It’s just something always there like I’m ex addict with hep C*, *do you know* … *like a leper* … *like some kind of diseased person”*. (Daniel, 41, on treatment)

Interestingly, a number of prisoners described a hierarchy of stigma in prison starting with being a drug user progressing to a PWID, an HCV infected drug user and finally a HIV infected drug user.

“*There should be more education in the prisons about it*, *for people who don’t need it*. *Because there is a stigma to it*, *you know what I mean*. *There’s a stigma to someone on gear*, *but there is a bigger stigma to someone on gear that used needles* …*that’s the way*, *and it’s like*, *people who use cocaine look down on someone who uses heroin*. *People who use heroin look down on someone that’s injecting heroin*. *Then people who are injecting heroin with hepatitis c look down on someone who has HIV and it’s just mad”*. (Raymond, 42, completed treatment with SVR)

Many of the study narratives included descriptions of feeling shame and stigma form an early age related to poverty and social status. No participant described stigma associated with a history of imprisonment as this was seen as normal within their peer group and the involvement in crime, particularly at any early stage was to fund the “right” items of clothing and “stuff” that allowed them to “fit in” with their better off peers.

“*Been judged and looked down on my whole life*. *All my life*, *since being a baby*, *being looked down you know*? *And then I get angry and then the resentment comes in*. *And I just remember cringing*, *the cringe*, *do you know what I mean*? *The shame*!*”*. (Michael, 35 deferred treatment)

### Positive experience of prison life and health care

The narratives reveal that prison life was often a much-needed break away from the chaos and competing priorities of their community lives. Incarceration was associated with the distress of being separated from loved ones, but also accepted as a normalised pattern of life. Imprisonment often provided prisoners with much needed stability.

“*I lost me Ma and Da and me other brother*. *Me brother was found* … *OD’ed* (overdosed) *as well*, *you know*, *so it just brought back memories*, *and I drifted back down*, *and I start taking crack* … *I was selling gear*, *and then I start selling crack at night*, *only getting half an hour sleep for months*, *and I got caught up with all the shit and I got locked up you know*. *This* (prison) *saved me if I’m being honest”*. (Kenneth, 41, completed treatment awaiting SVR)

All but two of the participants had linked with the in-reach hepatology nurses (specialist nurses who normally work in a hospital setting but provide HCV treatment at prison-based outpatient clinics). They describe this relationship as one of trust, familiarity, support and important in their HCV journey. The in-reach nurses where a constant presence over many re-incarcerations. Their level of engagement often reflected patients’ readiness to consider treatment and types of treatment available at the time. The issue of drug stability was a major factor during the interferon era with many guidelines and physicians excluding patients from HCV treatment on the grounds of active drug and alcohol use. The two participants who deferred treatment cited drug stability as the major factor in their decision. They both were engaging with addiction services and attempting to stabilise their drug dependence prior to starting HCV treatment.

“*Nurses build great relationships with prisoners and you see the nurses up there*, *they are second to none*. *They are brilliant* … *She gave me some amount of help up there*. *They are terrific and have a great rapport and a great respect”*. (Edward, 38, on treatment)

“*So*, *he’s been more or less there from the beginning* … *He’s been a good support to me*, *he doesn’t judge”*. (Nathan, 40, HCV co-infected and completed treatment)

### Transformative clinical and non-clinical changes associated with HCV treatment and cure

Those engaging in or completing treatment found DAA therapies easy to take, tolerable with minimal side effects. A few described experiencing insomnia and low energy levels in the early stages of treatment which resolved with basic management (reassurance and advice). The ease in which they completed treatment exceeded their expectations.

“*The only side effect I had was low energy* … *I couldn’t stop sleeping”*. (David, 42, on treatment)

“*I was a bit off the first week or two*. *Stomach cramps and sleep*. *But it kind of settled and I easily got through it”*. (Gary, 30, completed treatment with SVR)

“*A doddle*, *flew through it…way easier than expected”*. (Clint, 36, completed treatment with SVR)

Treatment was associated with increased energy, improved sleep, reduced pain and reduced abdominal distension. A number of participants reported a change in skin pallor, describing it as “whiter”, “less yellow” and “rejuvenated”.

“*I feel brilliant*. *All the swelling*, *I had a lot of swelling in my stomach and it’s all gone down*. *I’m sleeping properly; I’m not sweating as much*, *not getting pains as much*. *It really helped me”*. (Raymond, 42, completed treatment with SVR)

“*I noticed me eyes are white*, *after whitening up*. *Me energy*, *I have loads of energy*, *I’m doing the gym”*. (Paul, 52, completed treatment with SVR)

Many participants described positive transformative non-clinical outcomes from engagement with HCV treatment. Participants were visibly moved when speaking about these unexpected changes. Many spoke of “second chances”, “new beginnings” and “a new start”. These changes included: increased self-esteem, commitment to living a drug-free lifestyle, increased confidence and self-worth, identity and lifestyle changes including a reduction in substance misuse. These narratives were presented in the context of redemption and being grateful for having the opportunity to access the “expensive medication”.

“*Like at the moment I’m doing well*, *I’m off drugs five months*, *now*, *I’m drug free*, *there’s stuff on the landing and I even turned that down*, *so I’m happy- I’m after building up me willpower now*, *it’s a second chance for me and I can’t fuck it up”*. (Duane, 42, completed treatment with SVR)

“*I’m finished with drugs*. *I’ve been clean nearly a year*, *so I‘ll just be so careful*. *I even have dreams where I prick me self with a needle and I’m freaked out when I wake up and I’m sweating…”*. (Brendan, 59, completed treatment with SVR)

Many participants described the hope of social re-inclusion, including performing patient citizenship. They described the wish to return to work and care and provide for their families. Many hoped that this would be their last period of incarceration and expressed the need to make up for lost time.

“*I have a job there when I get out and all that* …*and I don’t want to fuck that up…When I’m going to leave the prison now* …*it was like getting the second chance in life to me*. *So*, *when I am leaving prison now*, *I have plans”*. (Thomas, 35, completed treatment with SVR)

“*It’s like*, *that was taken away from me*. *And it’s like*, *there’s another chance for it now if you want it*, *and I’ve never wanted to get clean as much as I do now*, *you know what I mean*. (Gavin, 37, on treatment)

A number of participants wished to engage in peer work. They spoke in terms of “giving back”. Some had benefited from peer workers while undergoing treatment and saw the benefits of this type of support. They also wanted to dispel the myths of treatment and to share their own positive experiences.

“*That’s the whole reason why I’m doing the interview* …*to help some other people*. *‘Hey*, *this isn’t as bad as what people make it out to be’ and encourage them to change their life around*, *just need the right tools*.” (Gary, 30, completed treatment with SVR)

### Reality of community release

Two of the study participants experienced community release during their prison sentence. Both had their temporary release suspended due to violations of its terms. Both describe a rapid return to high-risk drug taking and active dependence, mostly triggered by the stress of homelessness. Their narratives illustrate the lack of community support and structures available to prisoners on release and the complex psycho-social challenges experienced by both participants and their families. These relapses left them feeling hopeless to the point of contemplating self-harm. Both welcomed the return to prison and planned to get re-tested to check for re-infection and re-stabilise their drug use. One of the participants had acquired more criminal charges and was expecting a further sentence of three to five years.

“*It was the homelessness that got me*. *Not being able to cope out there*. *There were a couple of times that I was thinking about harming myself”*. (Clint, 36, completed treatment with SVR)

“*I had come off everything*, *got my head together*, *got out and things didn’t go too well on the outside* …*can’t really remember half the things that I was doing*, *cause I was taking tablets every day of the week and I ended up on the streets”*. (Duane, 42, completed treatment with SVR)

### Substantive theory

A grounded theory approach was utilised to produce a substantive theory of the need to increase the importance of HCV care among the routine competing priorities associated with the lives of PWID [32]. The collected narratives describe lives characterised by poverty, early school leaving and incarceration and high-risk opioid dependence associated with ongoing criminality and repeat incarcerations. Active dependence and homelessness remained the key priorities to manage while in the community and the relative stability of prison life, with access to trusted health care, increased the priority that prisoners assigned to their health care needs. Providing increased support to manage these multiple challenges, particular at locations where healthcare is delivered, has the potential to increase the priority given to health and HCV care. Imprisonment offers an opportunity to remove many of these identified challenges and engage hard-to-reach groups with healthcare. Furthermore, it is important the health care providers understand the challenges experienced by many socially marginalised groups and adapt a more understanding and user-friendly approach to how we provide healthcare: a one model approach does not fit all. For many HCV infection was still considered a relatively benign illness and treatment with feared historical treatments was not a consideration either in the community or while incarcerated. The availability of DAA treatments reduced these historical barriers and re-prioritised HCV care among the many challenges to be managed by HCV infected prisoners. Understanding and adapting this framework at a policy and healthcare delivery system level has the potential to improve HCV management. Expecting HCV infected PWID to engage with HCV care without contextualising it within the broader challenges of their daily lives is unreasonable. Adapting this framework for healthcare delivery both at an individual and population level has the potential to improve healthcare access and health outcomes for this and other marginalised and socially excluded populations.

“*I was diagnosed as a teenager … at first I didn’t understand about the consequences or anything… It’s only hep so I’ll be grand*. *I was*, *constantly abusing my body… using and sharing”*. (Leslie, 42, on treatment)

“*Because gear* (heroin) *was my main problem*, *Gear was my fucking number one problem”*. (Brendan, 59, completed treatment with SVR)

“*Some of us live a hectic life out there … no homes*, *off our heads on drugs … robbing*, *not everyone would be able to do it* (HCV treatment), *especially if they are still wrapped up in drugs*. *For me inside*, *it was the perfect opportunity”*. (Gavin, 37, on treatment)

“*Well it was good because it was a more stable environment (prison) … I was after building a good relationship with the hospital… and with SJ (hepatology nurse)*. *I was anxious and worried about…side effects…but there was none whatsoever*, *it was grand*, *there wasn’t a bother on me”*. (Emmett, 41, completed treatment with SVR)

## Discussion

The study reports unique findings from a qualitative study exploring prisoners’ experiences of their HCV journey from treatment to cure including: the experience of stigma and shame, the lack of relevance and importance assigned to HCV infection, competing priorities in the community, stability of prison life, trust in the in-reach hepatology services, ease of treatment and transformative clinical and non-clinical outcomes including increased self-esteem, commitment to living a drug-free lifestyle, increased confidence and self-worth, identity and lifestyle changes with the hope of social re-inclusion.

The barriers of knowledge deficit and fear of treatment described in this study have been reported previously[8,26]. For the study participants these were now historical since their experience of DAA treatment was less onerous and simpler than expected. These historical legacies of fear of treatment and its side effects still prevail and require interventions that increase information among high-risk groups. There is evidence that prison and community peer workers, in particular, those who have completed treatment, can increase uptake in HCV care and are seen as credible sources of information by prisoners[36,37]. The use of peer workers also fits with treated prisoners’ wishes to give back to their communities by sharing their experiences and encouraging and supporting others to engage with treatment.

The identification of stigma as a major barrier to PWID and prisoners engaging in healthcare, including HCV care, has been previously reported and was a consistent and repetitive theme across all narratives[8,10,38]. A number of prisoners who had successfully completed treatment still felt “contaminated”. The ongoing presence of HCV antibodies, even after treatment, can be confusing and in general, many cured patients still report having HCV infection[39]. This potentially maintains the stigma associated with HCV despite the patient successfully completing treatment[26].Using the language of cure and clearance may have a positive impact on prisoners and enhancing surveillance data to include more precise serological markers (HCV RNA status) may increase the understanding among those infected and the general public of the different stages of HCV infection[40]. In the long-term this may reduce the stigma linked with HCV infection with a greater understanding that being HCV RNA negative is indicative of cure and the ongoing presence of HCV antibody is historical. The stigma described by these study participants was not only linked with HCV infection and its association with IDU, it was also linked to poverty and a lifetime of marginalisation. Repeated incarceration from an early age was characteristic of this study cohort. Ongoing criminalisation of drug users ensures that this cycle continues and that our prisons remain overcrowded with the poorest and most marginalised in society[41–43]. Challenging existing drug policies of criminalisation and the ineffective “war on drugs”, is critical to tackling communicable diseases in prisons. At a broader level we need to support a range of public health policies (e.g. access to medical care including harm reduction services, housing and education) that improve the social and health inequalities experienced by many marginalised populations including prisoners[43,44].

It is recognised that in-reach hepatology nurses are a facilitator to HCV treatment both in community and prison settings [25,45,46]. A positive therapeutic relationship, based on trust and familiarity, increases treatment uptake and patient’s satisfaction and understanding of treatment[27]. Concerns have been expressed about how the ease of DAA treatment may erode some of the non-clinical benefits of HCV cure that were associated with interferon-based therapies[25]. The reduced clinical supervision required for DAA reduces nurse-patient contact and often eliminates the need to address the psycho-social issues that characterise prisoners’ lives[25]. This may impact on the therapeutic relationship between patient and treating nurse and while treatment may achieve HCV elimination, it may not address the other complex issues that can influence a return to drug use and drug-related risk behaviour.

The narratives collected in this study reveal the complex and overlapping relationship between the participants’ drug dependence and HCV journeys. All study participants had a history of IDU and all but one felt they had acquired HCV through the sharing of drug taking paraphernalia. Similar to other studies, active drug dependence was identified as a barrier to HCV care uptake and when present was prioritised among the many competing priorities[26,27]. Readiness is seen as an important factor in patient’s decision to engage in treatment[10]. This concept is closely linked with the cycle of change that underpins many peoples’ understanding of addictive behaviours and their treatment. [47]. Participants identified that achieving drug stability was a key priority and when achieved allowed them to contemplate addressing other issues including HCV care. While participant readiness was identified as a key factor to initiate in HCV therapy, practitioner support for engaging PWID into HCV-related care and structural support of PWID receiving access to reimbursed DAA therapies will also be needed to facilitate HCV treatment uptake among PWID[48,49]. The stability of prison life and access to addiction treatment, in particular MMT, and trusted healthcare further facilitated successful engagement in HCV care.

The clinical and non-clinical benefits of HCV treatment have been previously described[16,25,50]. This study reports similar outcomes including: improved energy, sleep and mood, reduced pain, sweating and abdominal swelling. These changes were associated with significant non-clinical transformations including increased self-esteem, self-confidence, sense of identity and a commitment to drug stability and not returning to prison. Linked with these changes was a hope of social re-inclusion, a return to work, family and community life and commitment to give back to their community by sharing their experiences to help others. There is potential to utilise this social capital for HCV prevention and strategy level by engaging treated prisoners in peer support networks and as recovery ambassadors and coaches (a non-clinical person who supports others recovering from drug dependence)[51,52]. It is important that expectations are managed since many challenges will still remain.

This study highlights the difficulty in maintaining stability on release. It is recognised that transition back to the community post-release is a challenging and high-risk period for prisoners[53–55]. It is also crucial to the management of HCV with linkage to care, addiction treatment services and harm reduction services a priority[53,56,57]. Return to homelessness and high-risk drug use characterised the experience of the two participants who had been released during their present sentence. These findings underscore the need for support on release and a commitment to addressing the social issues including homelessness experienced by prisoners released back into the community.

This study generated a substantive theory of increasing the importance of HCV care among the routine competing priorities associated with the lives of PWID. Reducing the impact of the many medical and social factors that impact PWID, can change the priority given to healthcare, including HCV treatment, in their lives[10,26]. This study identified drug dependence and homelessness as priority issues and the stability of prison life provides the opportunity to address these. Drug dependence, unemployment and homelessness have previously been identified as factors that impact negatively on the health and wellness and ability of PWID and released prisoners to access health care[58,59]. Homeless and marginalised people’s healthcare usage is characterised by delayed presentations, low usage of primary and preventative services, high usage of hospital emergency department (ED) and inpatient services and low usage of hospital outpatient department[60,61]. Recognising the competing priorities in the lives of HCV infected people should inform policy, health care delivery and HCV management. Providing additional supports alongside HCV treatment to address these complex needs could improve treatment uptake and outcomes. Providing HCV treatment at locations were HCV infected people attend for other health and social care needs (including prisons) could have a further positive impact on HCV management. The management of psycho-social and other health care needs (including addiction and mental health) often provides the time and capacity for PWID to manage other health care needs (including HCV care). Increasing the importance of health care among these challenges has the potential to increase uptake in HCV care. The substantive theory generated from this study can also inform further research on identifying barriers and facilitators to HCV screening and treatment uptake among PWID and prisoners.

The strengths of this study are the representativeness of those who participated. The one-to-one methodology allowed participants to reveal their HCV narratives in a private and confidential forum. A further strength of this study is the population studied. Prisoners are considered a hard to reach group and are rarely reported on in the published literature. There are a number of limitations to this study including the single gender and location of the study design. The interviewer was known to all the prisoners and provided addiction treatment to them during this or previous sentences. This may have prevented full disclosure and a biased narrative. Reversely, participants may have provided a more open and honest narrative to a researcher who was already known to them and who they had an existing therapeutic relationship with. This study did not analyse differences by treatment category and persons who have yet to receive treatment might have barriers that were not experienced by persons currently on treatment. Further research is warranted in this area.

## Conclusion

This study found that HCV infected Irish prisoners are characterised by early age of IDU (18.2 years) with high levels of needle sharing (72%) and drug paraphernalia sharing (100%). They are incarcerated from an early age (18.3 years) and experience repeat incarcerations, spending most of their adult lives in prison. Lifelong competing social and medical factors impact on their ability and willingness to engage in HCV treatment. The stability of prison life and access to familiar and trusted healthcare, in particular, in-reach hepatology nurses facilitated engagement with HCV care. Many HCV-infected prisoners have the disease for over a decade and suffered many associated physical and psychiatric morbidities. These were often only recognised by their improved wellness on the successful completion of treatment. Prisoners found DAA treatment easy to manage and describe a range of associated transformative non-clinical outcomes including: increased self-esteem, increased confidence and self-worth. These changes were often linked to a commitment to a drug free lifestyle and preventing HCV re-infection. Many expressed a hope of social re-inclusion including a commitment to engage with peers to educate and support them through treatment. Prison release is a high-risk and critical time for HCV infected prisoners who return to the competing priorities of community living. This is often associated with relapse into drug dependence and homelessness which quickly erodes the benefits achieved while incarcerated. Community support structures for recently released prisoners are lacking and this high-risk period for overdose deaths and HCV acquisition requires further research. The relevance of many of the themes identified in this study will reduce with the increasing commitment to HCV treatment delivery. However, the systemic issues of stigma, marginalisation, social and health inequalities and ongoing criminalisation of drug users will remain and will require Trojan efforts by many—including policymakers and politicians—to make meaningful change to those affected.

## Supporting information

S1 File Interview guide. (DOCX)
