# Supplementary material for: Competing priorities and second chances - A qualitative exploration of prisoners’ journeys through the Hepatitis C continuum of care
Source: PLoS One. 2019 Sep 11;14(9):e0222186. doi: 10.1371/journal.pone.0222186 (PMC6738615; doi:10.1371/journal.pone.0222186)
Supplement: S1 File — (DOCX) [file pone.0222186.s001.docx]

| **Area of Interest** | **Guiding Questions (Do not have to ask all questions if not required).** |
| --- | --- |
| **Demographics** | Age:  Gender: |
| **Hepatitis C Treatment** | Can you tell me about your HCV journey – when you were first diagnosed and where; how you might have acquired it; how did you know it was active; have you been offered treatment before; previous treatment?  What prevented you from seeking/getting/completing treatment before?  Acquisition of HCV- when and how?:  Length of time since HCV diagnosis:  Treatment yes/no:  Outcome:  SVR; yes/no/still on treatment/delayed starting treatment/referred to specialist services on release: |
| **Addiction Issues** | HX of IDU; yes/ no:  Age of first IV:  MMT; yes/no:  Length of time on MMT:  Other risk factors for HCV transmission:  Alcohol use- problem drinkers, every received treatment: |
| **Effects of Treatment** | How did going on treatment make you feel?  What has getting treatment for Hep C meant for you ?  (Prompts – tell me about any positive changes in your life as a result of getting access to this treatment? Tell me about any negative changes as a result of going on treatment?)  How did receiving the news that the Hepatitis C had been cured affect your life (IF APPLICABLE)?  (How did it affect your drug usage?) IF APPLICABLE  (Prompts how did it affect your injecting use .. sharing) IF APPLICABLE- |
| **The Treatment Programme.** | PRISON QUESTIONS  Tell me a bit about you HCV journey in prison – How often/ where were you tested?  Nurse/mass screening/ other?  Experience of (in-reach) Fibroscan  Experience of( in-reach hepatology  Experience of going to hospital  Experience of treatment  How has treatment impacted on your health?  Do you think you are at risk of re-infection?  What are the positives/negatives about screening and treatment in prison |
